# *Corynebacterium dentalis* sp. nov., a new bacterium isolated from dental plaque of a woman with periodontitis

**DOI:** 10.1016/j.nmni.2019.100625

**Published:** 2019-11-29

**Authors:** S. Benabdelkader, M. Boxberger, C.I. Lo, G. Aboudharam, B. La Scola, F. Fenollar

**Affiliations:** 1) Aix Marseille Université, IRD, AP-HM, MEФI, Marseille, France; 2) IHU-Méditerranée Infection, Marseille, France; 3) Aix Marseille Université, IRD, AP-HM, SSA, VITROME, Marseille, France; 4) UFR Odontologie, Aix-Marseille Université, Marseille, France

**Keywords:** bacteria, *Corynebacterium dentalis*, culturomics, dental plaque, taxono-genomics

## Abstract

Strain Marseille-P4122^T^ is a new species from the order *Corynebacteriales* that was isolated from the dental plaque of a woman with periodontitis. It is a facultative anaerobic Gram-positive rod-shaped bacterium. Strain Marseille-P4122^T^ exhibited a 98.19% sequence identity with *Corynebacterium suicordis* strain P81/02, the phylogenetically closely related species with standing in nomenclature. The draft genome size of strain Marseille-P4122^T^ is 2.49 Mb with 60.1% G + C content. We propose that strain Marseille-P4122^T^ (=CSURP4122) is the type strain of the new species *Corynebacterium dentalis* sp. nov.

## Introduction

*Corynebacterium* genus belonging to family *Corynebacteriaceae* was first described in 1896 by Lehmann and Neumann [1]. It consists of Gram-positive rods and non-spore-forming bacteria with a high DNA G + C content [2]. Several species of this genus are implicated in human and animal diseases whereas others are members of normal flora on skin and mucous membranes [[3], [4], [5]]. *Corynebacterium diphtheriae* is the major pathogen in humans and causes diphtheria worldwide [6]. It is a large genus that regroups currently 132 species with 11 subspecies validly described with standing in nomenclature [7].

It is important to understand the implications of bacterial diversity in normal physiological functions and for disease [8]. Culturomics is a concept that develops different culture conditions to enlarge our knowledge of the human microbiota through the discovery of previously uncultured bacteria [[9], [10], [11], [12]]. Once a bacterium is isolated, we use a taxono-genomics approach including matrix-assisted laser desorption/ionization time-of-flight mass spectrometry (MALDI-TOF MS), phylogenetic analysis, main phenotypic description and genome sequencing, to describe it [13,14].

Here we describe *Corynebacterium dentalis* sp. nov., strain Marseille-P4122^T^ (=CSUR P4122), following this taxono-genomics concept.

## Isolation and growth conditions

**Fig. 1:**
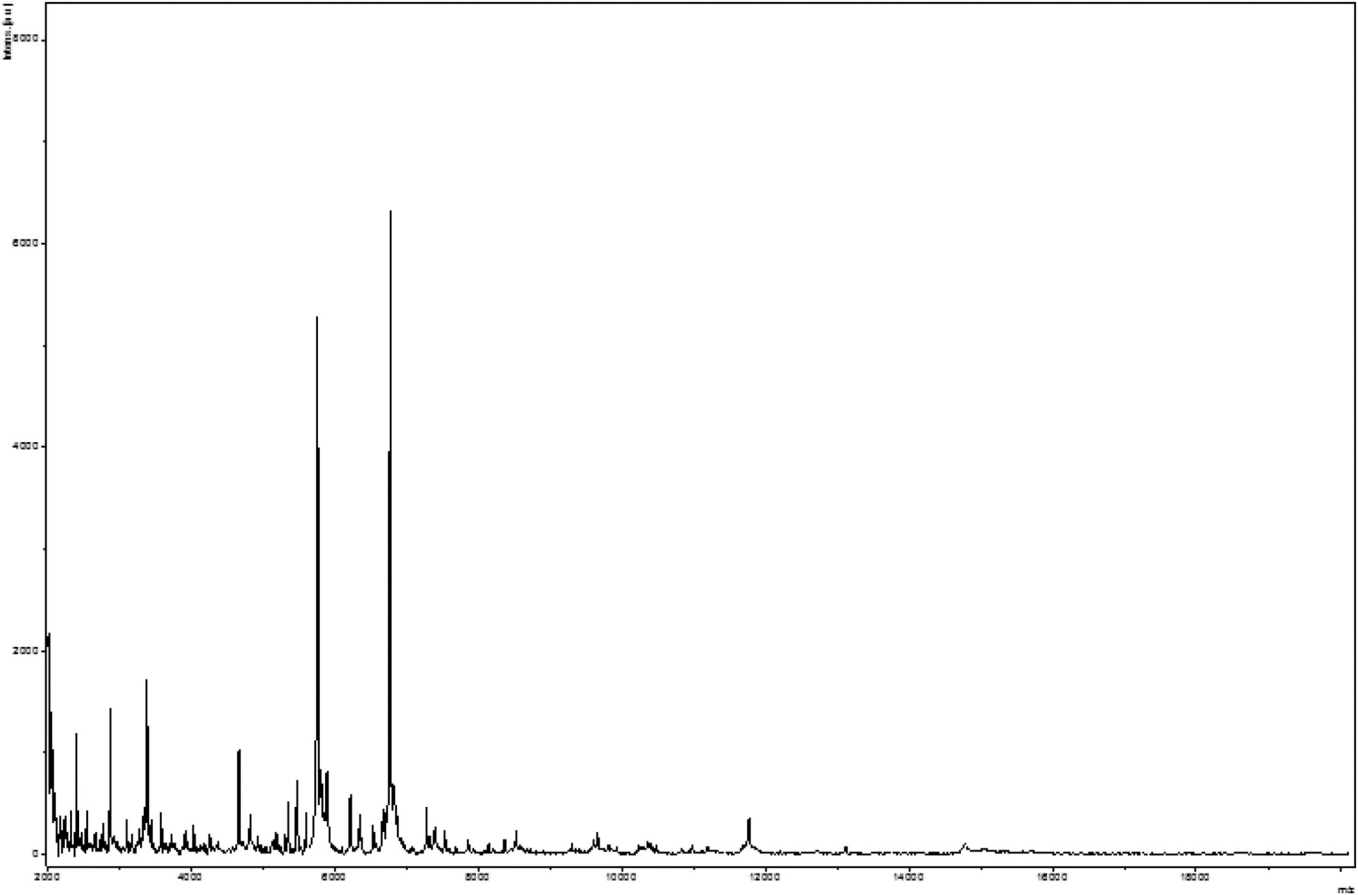
MALDI-TOF MS reference mass spectrum of *Corynebacterium dentalis* sp. nov., strain Marseille-P4122T. The reference spectrum was generated by comparison of spectra from 12 individual colonies.

In 2015, we isolated from the dental plaque sample of a woman with periodontitis an unidentified bacterial strain. A screening was performed using MALDI-TOF MS on a Microflex LT spectrometer (Bruker Daltonics, Bremen, Germany) as previously described [15]. The spectra obtained (Fig. 1) were imported into MALDI Biotyper 3.0 software (Bruker Daltonics) and analysed against the main spectra of the bacteria included in two databases (Bruker and the constantly updated MEPHI databases). The study was validated by the ethics committee of the Institut Fédératif de Recherche IFR48 under number 2016-010. Strain Marseille-P4122^T^ was first isolated in aerobic conditions after incubation in a culture bottle (bioMérieux, Marcy l’Etoile, France) supplemented with 5 mL sheep blood at 37°C.

## Phenotypic characteristics

**Fig. 2:**
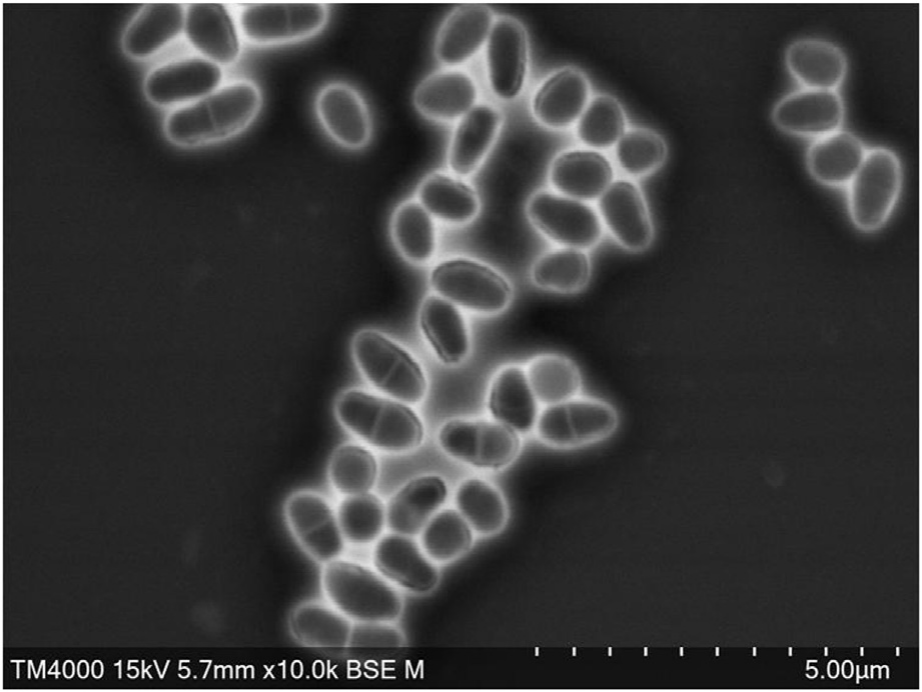
Scanning electron microscopy of stained *Corynebacterium dentalis* sp. nov., (Hitachi TM4000). Scales and acquisition settings are shown on the figure.

After the isolation step, the strain Marseille-P4122^T^ was cultured to obtain pure and isolated colonies on blood agar. The colonies were white and transparent. Bacterial cells were Gram-positive. The sporulation test (10 min at 80°C) was negative. Different growth temperatures (20, 28, 32, 37, 45 and 56°C), pH (5, 6, 7, 7.5, 8 and 8.5), NaCl content (5, 10 and 15 g/L) and atmospheres (aerobic, anaerobic and microaerophilic (CampyGEN; Oxoid, Basingstoke, UK)) were tested on 5% sheep-blood-enriched Columbia Agar. Strain Marseille-P4122^T^ is a very-easy-to-cultivate bacterium and grows in all these conditions except at 56°C. API ZYM and API Coryne tests (bioMérieux) were performed to determine specific phenotypic features for strain Marseille-P4122. The results are shown in Table 1. Using API 50CH strips (bioMérieux) the carbohydrate metabolism of strain Marseille-P4122 was evaluated according to the manufacturer's instructions (Table 2). Strain Marseille-P4122^T^ has enzymatic activities such as esterase (C4), esterase-lipase (C8), lipase (C14), acid phosphatase, naphthol-AS-BI-phosphohydrolase, α-glucosidase, β-glucosidase and urease, whereas only d-fructose and d-trehalose were positive for carbohydrate metabolism. All the other reactions tested were negative. Strain Marseille-P4122^T^ showed catalase-negative and oxidase-negative activities. A comparative study of the biochemical characteristics of this strain with other closely related *Corynebacterium* species is presented in Table 3. For scanning electron microscopy, a colony was collected from agar and immersed into a 2.5% glutaraldehyde fixative solution. The slide was gently washed in water, air-dried and examined with a TM4000 microscope. The cells appeared rod-shaped with a mean length of 1 μm and a mean diameter of 0.5 μm (Fig. 2). Antimicrobial susceptibility testing was performed using the E-test strips (bioMérieux) method and the data obtained are summarized in Table 4. The major fatty acids found for this strain were hexadecanoic acid (44%) and 9-octadecenoic acid (36%). Very few other structures were described. No branched fatty acids were detected (Table 5).

**Table 1:** Phenotypic characterization of *Corynebacterium dentalis* sp. nov., based on analytical profile index (API) ZYM and CORYNE tests

Tests	Characteristics	Results
API ZYM	Alkaline phosphatase	–
	Esterase (C4)	+
	Esterase lipase (C8)	+
	Lipase (C14)	+
	Leucine arylamidase	–
	Valine arylamidase	+
	Cystine arylamidase	–
	Trypsin	–
	α-Chymotrypsin	–
	Acid phosphatase	–
	Naphthol-AS-BI-phosphohydrolase	+
	α-Galactosidase	+
	β-Galactosidase	–
	β-Glucuronidase	–
	α-Glucosidase	–
	β-Glucosidase	–
	*N*-Acetyl-β-glucosaminidase	–
	α-Mannosidase	–
	α-Fucosidase	–
	Glycerol	–
API CORYNE	Nitrate reductase	–
	Pyrazinamidase	–
	Pyrrolidonyl arylamidase	–
	Alkaline phosphatase	+
	β-Glucuronidase	–
	β-Galactosidase	–
	α-Glucosidase	–
	*N*-Acetyl-β-glucosaminidase	–
	β-Glucosidase	–
	Urease	+
	Gelatin	–
	Control	–
	d-Glucose	–
	d-Ribose	–
	d-Xylose	+
	d-Mannitol	–
	d-Maltose	–
	d-Lactose	+
	d-Saccharose	–
	Glycogen	+

**Table 2:** Phenotypic characterization of *Corynebacterium dentalis* sp. nov., based on API 50 CH test

Tests	Characteristics	Results
50 CH	Erythritol	–
	d-Arabinose	–
	l-Arabinose	–
	d-Ribose	–
	d-Xylose	–
	l-Xylose	–
	d-Adonitol	–
	Methyl βd-xylopyranoside	–
	d-Galactose	–
	d-Glucose	–
	d-Fructose	+
	d-Mannose	–
	l-Sorbose	–
	l-Rhamnose	+
	Dulcitol	–
	Inositol	–
	d-Mannitol	–
	d-Sorbitol	–
	Methyl αd-mannopyranoside	–
	Methyl αd-glucopyranoside	–
	*N*-Acetyl-glucosamine	–
	Amygdalin	–
	Arbutin	–
	Esculin ferric citrate	–
	Salicin	–
	d-Cellobiose	–
	d-Maltose	–
	d-Lactose	–
	d-Melibiose	–
	d-Saccharose	–
	d-Trehalose	+
	Inulin	–
	d-Melezitose	–
	d-Raffinose	–
	Amidon	–
	Glycogen	–
	Xylitol	–
	Gentiobiose	–
	d-Turanose	–
	d-Xylose	–
	d-Tagalose	–
	d-Fucose	–
	l-Fucose	–
	d-Arabitol	–
	l-Arabitol	–
	Potassium gluconate	
	Potassium 2-ketogluconate	–
	Potassium 5-ketogluconate	–

**Table 3:** Comparison of differential characteristics between *Corynebacterium dentalis* sp. nov., and other bacterial species, *Corynebacterium resistens*, *Corynebacterium suicordis*, *Corynebacterium urinapleomorphum* and *Corynebacterium phoceense*

Property	*C. dentalis*	*C. resistens*	*C. suicordis*	*C. urinapleomorphum*	*C. phoceense*
Cell diameter (μm)	0.5	NA	NA	0.2	0.5
Oxygen requirement	+	±	±	+	+
Gram stain	+	+	+	+	+
Salt requirement	–	–	–	–	–
Motility	–	–	–	–	–
Endospore formation	–	–	–	–	+
Alkaline phosphatase	–	+	+	+	+
Catalase	–	+	+	+	+
Oxidase	–	–	–	–	–
Nitrate reductase	–	–	–	NA	+
Urease	+	–	+	+	–
β-Galactosidase	–	–	–	–	–
*N*-Acetyl-glucosamine	–	–	–	–	–
Arabinose	–	–	–	–	NA
Lipase (C8)	+	+	+	+	+
Pyrrolidonyl arylamidase	–	+	+	–	+
Mannose	–	–	–	–	+
Mannitol	–	–	–	–	–
Sucrose	NA	–	–	NA	–
d-Glucose	–	+	–	–	+
d-Fructose	+	–	–	–	+
d-Maltose	–	–	–	–	+
Source	Human	Human	Pig	Human	Human

**Table 4:** Sensitivity test to certain antibiotics on the strain Marseille-P4122^T^

Antibiotics used	MIC (mm)	Reference values	Interpretations
Rifampicin	0.003	≤0.06 to >0.5	Susceptible
Ciprofloxacin	0.064	<0.06 to >0.5	Susceptible
Daptomycin	0.094	<0.25 to >0.5	Susceptible
Amoxicillin	0.125	≤0.25 to ≥1	Susceptible
Penicillin G	0.19	<0.06 to >0.5	Susceptible
Doxycycline	0.38	≤0.12 to ≥0.5	Susceptible
Vancomycin	0.38	≤2 to >2	Susceptible
Erythromycin	16	≤0.5 to ≥8	Resistant
Imipenem	0.023	≤2 to ≥8	Susceptible
Amikacin	0.5	≤4 to ≥16	Susceptible

**Table 5:** Fatty acid profiles (%) of *Corynebacterium dentalis* strain Marseille-P4122^T^

Fatty acids	Name	Mean relative %∗
16:00	Hexadecanoic acid	44.2 ± 1.5
18:1n9	9-Octadecenoic acid	35.6 ± 1.0
18:00	Octadecanoic acid	9.3 ± 0.5
18:2n6	9,12-Octadecadienoic acid	5.9 ± 0.3
17:00	Heptadecanoic acid	4.5 ± 0,3
14:00	Tetradecanoic acid	TR

∗ Mean peak area percentage; TR, trace amounts <1%.

## Strain identification

**Fig. 3:**
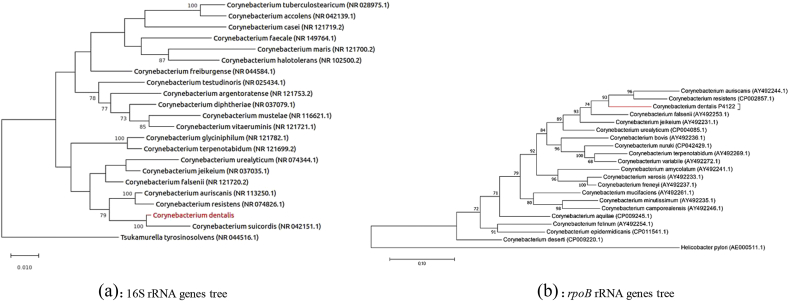
Phylogenetic trees highlighting the position of *Corynebacterium dentalis* sp. nov., based on the 16S rRNA gene sequences (a) and the rpoB gene sequences (b) relative to the most closely related type strains within the genus *Corynebacterium*. GenBank accession numbers are indicated in parentheses. Sequences were aligned using MUSCLE with default parameters, phylogenetic inference were obtained using the maximum likelihood method and the MEGA 7 software. Numbers at the nodes are percentages of bootstrap values obtained by repeating the analysis 1000 times to generate a majority consensus tree. The scale bar indicates a 1% nucleotide sequence divergence.

The 16S rRNA gene was sequenced to classify this bacterium. Amplification was carried out using the primer pair fD1 and rP2 (Eurogentec, Angers, France) and sequencing using the Big Dye® Terminator v1.1 Cycle Sequencing Kit and 3500xL Genetic Analyzer capillary3500xL sequencer (Thermofisher, Saint-Aubin, France), as previously described [16]. The 16S rRNA nucleotide sequences were assembled and corrected using CodonCode Aligner software (http://www.codoncode.com). Strain Marseille-P4122^T^ exhibited a 98.19% sequence identity with *Corynebacterium suicordis* strain P81/02 (GenBank accession number NR042151.1), the phylogenetically closest species with standing in nomenclature (Fig. 3a). The *rpoB* gene that encodes the β subunit of bacterial RNA polymerase was targeted to discriminate the *Corynebacterium* species [17]. *Corynebacterium dentalis* strain Marseille-P4122^T^ was close to strains *Corynebacterium auriscanis* and *Corynebacterium resistens* (Fig. 3b). Considering these phylogenetic criteria, we consequently classify this strain as a member of a new species within the genus *Corynebacterium*, family *Corynebacteriaceae*, phylum Actinobacteria.

## Genome sequencing

**Fig. 4:**
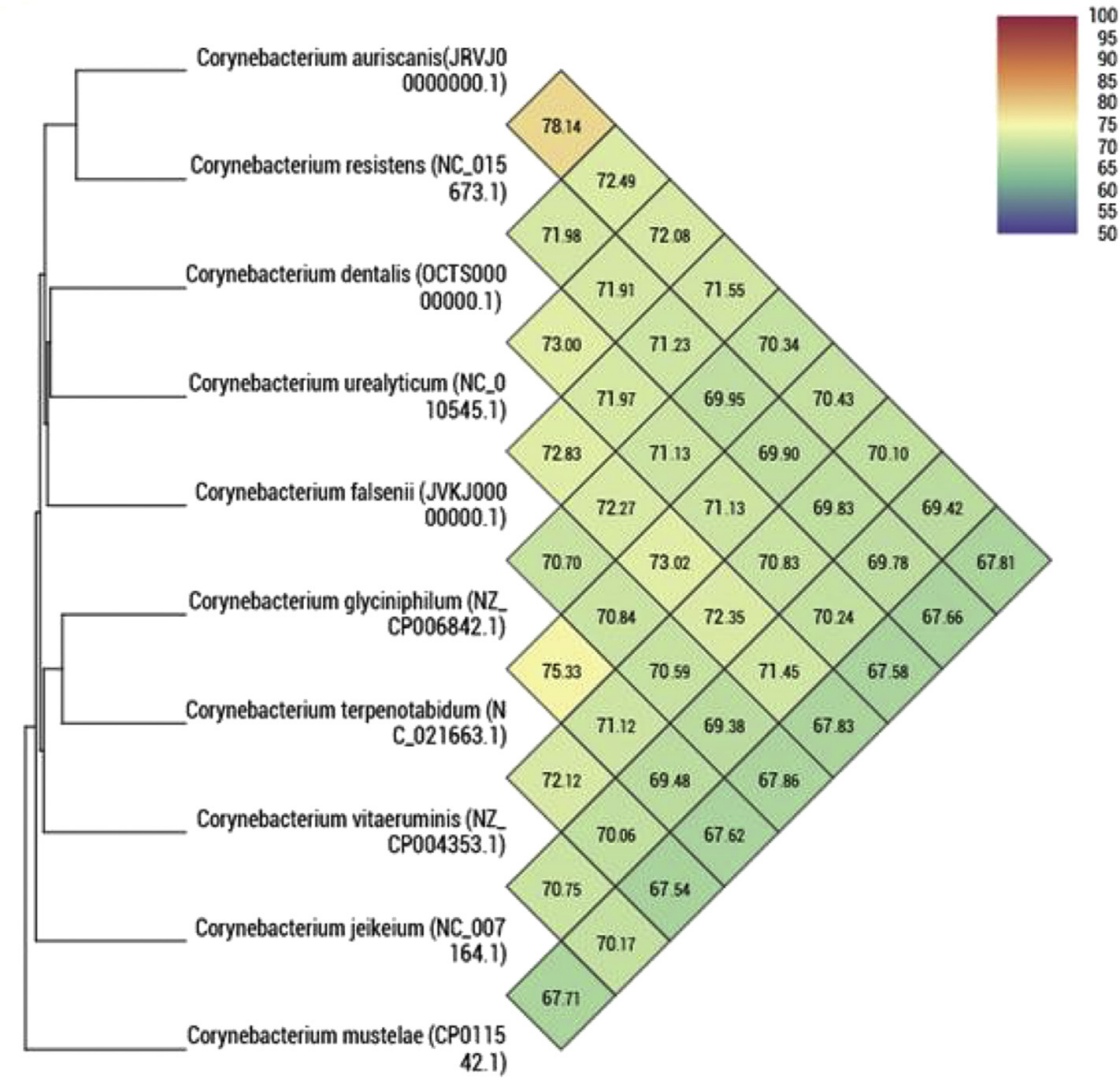
Heatmap generated with OrthoANI values calculated using the OAT software between *Corynebacterium dentalis* sp. nov., and other closely related species with standing in nomenclature.

Genomic DNA was extracted using the EZ1 biorobot (Qiagen, Courtaboeuf, France) with the EZ1 DNA tissue kit and then sequenced on the MiSeq technology (Illumina, San Diego, CA, USA) with the Nextera Mate Pair sample prep kit and Nextera XT Paired end (Illumina), as previously described [18]. The assembly was performed with a pipeline incorporating different softwares (Velvet [19], Spades [20] and Soap Denovo [21]), and trimmed data (MiSeq and Trimmomatic [22] softwares) or untrimmed data (only MiSeq software). GapCloser was used to reduce assembly gaps. Scaffolds <800 bp and scaffolds with a depth value < 25% of the mean depth were removed. The best assembly was selected using different criteria (number of scaffolds, N50, number of N). The genome of *Corynebacterium dentalis* strain Marseille-P4122^T^ is 2 303 041 bp long with a 60.1% G + C content. The degree of genomic similarity of strain Marseille-P4122^T^ with closely related species was estimated using the OrthoANI software [23]. Values among closely related species (Fig. 4) ranged from 75.33% between *Corynebacterium glyciniphilum* and *Corynebacterium terpenotabidum* to 78.14% between *Corynebacterium auriscanis* and *Corynebacterium resistens*. When the isolate was compared with these closely related species, values ranged from 67.54% with *Corynebacterium vitaeruminis* and *Corynebacterium jeikeium* to 78.14% with *Corynebacterium auriscanis*.

## Conclusion

Based on the results from unique phenotypic characteristics, including API galleries tests, MALDI-TOF spectrum, and phylogenetic and genomic analysis such as 16S rRNA sequence similarity <98.7% and OrthoANI value < 95% with the phylogenetically closest species with standing in nomenclature, we formally proposed strain Marseille-P4122^T^ as the type strain of *Corynebacterium dentalis* sp. nov.

## Description of Corynebacterium dentalis sp. nov.

*Corynebacterium dentalis* (den.ta'lis. N.L. masc. adj. *dentalis* referring to the teeth surrounded by dental plaque from which this strain was isolated). The strain grows easily in varied conditions. Optimum growth of colonies was obtained at 37°C on 5% sheep-blood-enriched Columbia Agar in <24 hours. They appear white and transparent. *Corynebacterium dentalis* is a Gram-positive rod-shaped bacterium with a mean length of 1 μm and a mean diameter of 0.5 μm. Strain Marseille-P4122^T^ produced esterase, lipase, acid phosphatase, naphthol-AS-BI-phosphohydrolase, α-glucosidase, β-glucosidase, urease, d-fructose and d-trehalose. But no activity was observed with trypsin, β-galactosidase, α-glucosidase, glycerol, d-arabinose, d-ribose, d-xylose, d-glucose, d-fructose, d-mannose, l-rhamnose, d-lactose, d-saccharose, glycogen, d-fucose and d-arabitol. Strain Marseille-P4122^T^ is catalase-negative. It is susceptible to rifampicin, ciprofloxacin, amoxicillin, penicillin G, doxycycline and vancomycin, but resistant to erythromycin. The genome size of *Corynebacterium dentalis* strain Marseille-P4122^T^ is about 4.04 Mb with 60.1 mol% G + C content. The GenBank Accession number for the 16S rRNA gene sequence of strain Marseille-P4122^T^ is LT897837 and for the whole-genome shotgun project is OCTS00000000.This strain was isolated from the dental plaque of a woman with periodontitis.

## Nucleotide sequence accession number

The 16S rRNA gene and genome sequences were deposited in GenBank under accession numbers LT897837 and OCTS00000000, respectively.

## Deposit in culture collections

Strain Marseille-P4122^T^ was deposited in our strain collections under number (=CSURP4122).

## Funding sources

This study was supported by the Institut Hospitalo-Universitaire (IHU) Méditerranée Infection, the National Research Agency under the program«Investissements d'avenir», reference ANR-10-IAHU-03, the Région Provence Alpes Côte d’Azur and European funding FEDER PRIMI.

## Conflict of interest

None to declare.
